# Retraction Note: RNAi targeting CXCR4 inhibits proliferation and invasion of esophageal carcinoma cells

**DOI:** 10.1186/s13000-019-0828-y

**Published:** 2019-05-20

**Authors:** Tao Wang, Yanfang Mi, Linping Pian, Ping Gao, Hong Xu, Yuling Zheng, Xiaoyan Xuan

**Affiliations:** 1 Department of Hemato-tumor, The First Affiliated Hospital of Henan College, University of TCM, Zhengzhou, People’s Republic of China; 2 The First Affiliated Hospital of Zhengzhou University, Zhengzhou, 450052 People’s Republic of China; 3 Department of Otolaryngology Head and Neck Surgery, The Second Affiliated Hospital of Zhengzhou University, Zhengzhou, People’s Republic of China; 4 Department of Ultrasound, The First Affiliated Hospital College, TCM of Henan, Zhengzhou, People’s Republic of China; 5 Henan Tumor Institute, Zhengzhou, People’s Republic of China; 6 Henan University of TCM, Zhengzhou, People’s Republic of China; 7 College of Basic Medical Sciences, Zhengzhou University, Zhengzhou, People’s Republic of China

**Retraction Note: Diagnostic Pathology (2013) 8:104**

**https://doi.org/10.1186/1746-1596-8-104**

The Editor-in-Chief has retracted this article [1] because Fig. 3 shows overlap with Fig. 6 in [2], Fig. 2b in [3] and Fig. 6a in [4]. An investigation by Zhengzhou University has confirmed that these figures overlap. The data reported in this article are therefore unreliable.

All authors have agreed to this retraction.
